# Development and characterization of a novel C-terminal inhibitor of Hsp90 in androgen dependent and independent prostate cancer cells

**DOI:** 10.1186/1471-2407-11-468

**Published:** 2011-10-31

**Authors:** Jeffery D Eskew, Takrima Sadikot, Pedro Morales, Alicia Duren, Irene Dunwiddie, Megan Swink, Xiaoying Zhang, Stacey Hembruff, Alison Donnelly, Roger A Rajewski, Brian SJ Blagg, Jacob R Manjarrez, Robert L Matts, Jeffrey M Holzbeierlein, George A Vielhauer

**Affiliations:** 1 Department of Urology, The University of Kansas Medical Center, 3901 Rainbow Blvd, Kansas City, KS, 66160, USA; 2 The University of Kansas Cancer Center, The University of Kansas Medical Center, 3901 Rainbow Blvd, Kansas City, KS, 66160, USA; 3 Department of Pharmaceutical Chemistry, The University of Kansas, 2095 Constant Ave, Lawrence, KS, 66047, USA; 4 Department of Medicinal Chemistry, The University of Kansas, 1251 Wescoe Hall Drive, Lawrence, KS, 66045, USA; 5 Department of Biochemistry and Molecular Biology, Oklahoma State University, 246C Noble Research Center, Stillwater, OK, 74078, USA

**Keywords:** Hsp90, prostate cancer, novobiocin, C-terminal inhibitors, N-terminal inhibitors

## Abstract

**Background:**

The molecular chaperone, heat shock protein 90 (Hsp90) has been shown to be overexpressed in a number of cancers, including prostate cancer, making it an important target for drug discovery. Unfortunately, results with *N-*terminal inhibitors from initial clinical trials have been disappointing, as toxicity and resistance resulting from induction of the heat shock response (HSR) has led to both scheduling and administration concerns. Therefore, Hsp90 inhibitors that do not induce the heat shock response represent a promising new direction for the treatment of prostate cancer. Herein, the development of a C-terminal Hsp90 inhibitor, KU174, is described, which demonstrates anti-cancer activity in prostate cancer cells in the absence of a HSR and describe a novel approach to characterize Hsp90 inhibition in cancer cells.

**Methods:**

PC3-MM2 and LNCaP-LN3 cells were used in both direct and indirect *in vitro *Hsp90 inhibition assays (DARTS, Surface Plasmon Resonance, co-immunoprecipitation, luciferase, Western blot, anti-proliferative, cytotoxicity and size exclusion chromatography) to characterize the effects of KU174 in prostate cancer cells. Pilot *in vivo *efficacy studies were also conducted with KU174 in PC3-MM2 xenograft studies.

**Results:**

KU174 exhibits robust anti-proliferative and cytotoxic activity along with client protein degradation and disruption of Hsp90 native complexes without induction of a HSR. Furthermore, KU174 demonstrates direct binding to the Hsp90 protein and Hsp90 complexes in cancer cells. In addition, in pilot *in-vivo *proof-of-concept studies KU174 demonstrates efficacy at 75 mg/kg in a PC3-MM2 rat tumor model.

**Conclusions:**

Overall, these findings suggest C-terminal Hsp90 inhibitors have potential as therapeutic agents for the treatment of prostate cancer.

## Background

Prostate cancer is generally recognized as a relatively heterogeneous disease lacking strong biological evidence to implicate specific oncogenesis, mutations, signaling pathways, or risk factors in tumorigenesis and/or resistance to therapy across patients. In 1952, Huggins and Hodges first reported susceptibility of prostate cancer to androgen withdrawal. Since that time, hormonal therapy has become a mainstay for prostate cancer treatment; however, despite dramatic initial clinical responses, virtually all patients ultimately fail androgen-targeted ablation. Experimental therapies in prostate cancer such as targeted agents, immunotherapy, and vaccine therapy exhibit limited efficacy and no improvement in survival [1]. Thus, a critical need for novel therapies to treat prostate cancer remains.

One such approach is based on the development of small molecules that inhibit Hsp90 chaperone function which leads to the degradation of Hsp90 dependent oncogenic proteins, many of which are involved in a multitude of signaling cascades. Inhibitors of Hsp90 (Hsp90-I) effect numerous proteins and pathways that are critical to the etiology of prostate cancer [2-4] and have demonstrated significant anti-proliferative effects in multiple cancer models, many of which are being evaluated in clinical trials [5]. To date, most Hsp90-I are N-terminal inhibitors. One example is the geldanamycin derivative, 17-allylamino-17-demethoxygeldanamycin (17-AAG). 17-AAG has demonstrated promising preclinical activity *in-vitro *and *in-vivo *[6-8]. Unfortunately, like other N-terminal inhibitors, the efficacy of 17-AAG is hampered by the fact that Hsp90 inhibition itself initiates a heat shock response (HSR), ultimately resulting in the induction of Hsp90 and anti-apoptotic proteins such as Hsp70 and Hsp27 [9-11]. Furthermore, induction of Hsp70 has been linked to chemoprotection [12-14]. In fact, the largely cytostatic profile observed upon administration of 17-AAG across cancers is likely the result of the pro-survival Hsp induction. This is supported by studies showing that neutralizing Hsp72 and Hsp27 activity or their transcriptional inducer, HSF-1 augments the effect of 17-AAG and dramatically increases the extent of apoptosis [11,15,16]. Others have shown that combinatorial approaches consisting of 17-AAG and transcriptional inhibition of pro-survival Hsp's improves the efficacy of 17-AAG [17].

In contrast to N-terminal inhibitors, the coumarin antibiotic novobiocin (NB) binds to the C-terminus of Hsp90, inhibits its activity, but does not elicit a HSR [18,19]. Previously the synthesis, screening and characterization of NB analogues has been reported and have demonstrated that molecules can be synthesized to exhibit improved potency relative to NB [18,20,21]. Interestingly, depending on the side-chain substitution of the coumarin ring, these NB analogues can manifest potent anti-proliferative and cytotoxic effects with minimal Hsp induction or demonstrate neuroprotective effects in the absence of cytotoxicity [18,19,22]. Herein, the distinct biological activity of the second generation analog, KU174 is described. KU174 demonstrates relative selective and rapid cytotoxicity (6 hr) along with client protein degradation in the absence of a HSR in hormone dependent and independent prostate cancer cell lines. Additionally, this work extends our understanding of the biology and mechanism of C-terminal inhibition by characterizing native chaperone complexes using Blue-Native (BN) electrophoresis and size exclusion chromatography (SEC). Under these native conditions, distinct responses are observed to the Hsp90α, Hsp90β, and GRP94 complexes following treatment with KU174 including the degradation of Hsp90β. Furthermore, the direct binding of KU174 to recombinant Hsp90 is described along with the functional inhibition of Hsp90 using a novel cell-based Hsp90-dependent luciferase refolding assay. Finally, the *in vivo *efficacy and selective tumor uptake of KU174 is reported in a pilot rat PC3-MM2 xenograft tumor study.

## Methods

NB analogues were synthesized as previously described [23]. F-4, KU-174, NB and 17-AAG were dissolved in DMSO and stored at -80°C until use. Commercial antibodies were obtained for Hsp90 isoforms (α/β), Hsc70, GRP94 (Santa Cruz Biotechnology, Inc., Santa Cruz, CA), Hsp27, Hsp70, HSF1, survivin, Akt, Caspase-3, Her2/Erb2, HOP, Actin (Cell Signaling Technologies, Danvers, MA), and Hsp60 (Epitomics, Inc., Burlingame, CA).

### Cell line acquisition and authentication

All cells were obtained from ATCC (Manassas, VA). Prior to manuscript submission, genomic DNA from frozen stocks of cell lines were submitted for short tandem repeat analysis [24] at RADIL (University of Missouri). Profiling results for each cell line were compared to those listed on the ATCC website.

### Cell culture

PC3-MM2-MM2 (androgen independent) and LNCaP-LN3 (androgen dependent) prostate cancer cell-lines [25] were obtained from M.D. Anderson Cancer Center (Houston, TX) and cultured in MEM Eagle media (Sigma-Aldrich, St. Louis, MO), respectively, with 10% FBS and penicillin/streptomycin (100 IU/ml/100 mg/ml) and maintained at 37°C with 5% CO_2_. Freeze downs stocks of the original characterized cell-line were stored under liquid nitrogen. All experiments were performed using cells with < 20 passages and < three months in continuous culture. Normal human renal proximal tubule epithelial cells (RPTEC) were purchased from Clonetics (Walkersville, MD) and grown per manufacturer instructions. RPTEC cells were not passaged more than six times.

### NCI Anti-proliferation Experiments of the NCI panel of 60 Cancer Cell lines

NCI60 tumor cell line screen was conducted by the Developmental Therapeutics Program at NCI (http://dtp.cancer.gov/) and was performed as previously described [26]. Briefly, KU174 was run in a five concentration dose response against the NCI panel of 60. From dose response curves, growth inhibition of 50% (GI50) was calculated from [(Ti-Tz)/(C-Tz)] × 100 = 50, which is the drug concentration resulting in a 50% reduction in the net protein increase (as measured by SRB staining) in control cells during the drug incubation.

### Annexin V apoptosis experiments

Cells were stained for Annexin V and propidium iodide (PI) as previously described [18] and according to the manufacturer's instructions (Invitrogen, Carlsbad, CA). The data displayed represented the mean SEM of three independent experiments (n = 3).

### Trypan blue cytotoxicity experiments

Cell viability was conducted as previously described [18]. Briefly, at the end of the incubation time for each cell treatment group, non-adherent cells were collected and combined with cells detached by trypsinization using TrypLE™ Express (Invitrogen, Carlsbad, CA) followed by centrifugation at 200 × g at 4°C. Cell pellet was then re-suspended and washed twice with cold DPBS (Invitrogen, Carlsbad). Total cell counts and viability was conducted on an automated system Vi-Cell™, Beckman Coulter, Inc., Brea, CA).

### Western blot

PC3-MM2 or LNCaP-LN3 cells were seeded at a density of 1.5 × 10^6 ^in T75 flasks. After 24 hours the T = 0 flask was harvested and cells counted by Vi-Cell. Remaining flasks were dosed with drugs by serial dilution from DMSO stocks. Total cells after 24 hours were pelleted and suspended into PBS. Suspended cells were aliquoted for Vi-Cell cell viability measurements, total protein SDS-PAGE analysis and Blue-native (BN) electrophoresis. SDS-PAGE lysates were prepared in RIPA (50 mM Tris-HCl pH 7.5, 150 mM, containing 0.1% SDS, 1% Igepal, 1% sodium deoxycholate, protease and phosphatase inhibitor cocktail, Sigma-Aldrich, Inc., St. Louis, MO) and lysed by three freezing and thawing cycles using liquid nitrogen and 37°C water bath. Protein concentration was determined using DC Protein Assay (Bio-Rad Laboratories, Hercules, CA) and a total of 25 μg of cell lysates were used for Western blot.

### Blue-native gel electrophoresis

BN lysates were prepared from PC3-MM2 or LNCaP-LN3 cells in 20 mM Bis-Tris (pH 7.4), 125 mM caproic acid, 20 mM KCl, 2 mM EDTA, 5 mM MgCl_2_, 10% glycerol and 2% n-dodecyl beta-D-maltoside (DDM) followed by three freezing and thawing cycles and centrifugation at 14,000 × g for 30 min at 4° C. Protein concentration was determined as described above and equal amounts of protein loaded on a Native PAGE Novex 3-12% Bis-tris gel (Invitrogen, Carlsbad, CA) and electrophoresed according to manufacturer's instructions.

### Size exclusion chromatography (SEC)

BN cells lysates, prepared as described above, were injected onto a HiPrep 16/60 Sephacryl S-300 column. SEC running buffer contained 20 mM Bis-Tris (pH 7.4), 125 mM caproic acid, 20 mM KCl, 2 mM EDTA, 5 mM MgCl_2_, and 10% glycerol. Chromatography was performed on an ATKAprime plus (GE Healthcare) at 0.5 mL/min and fractions (0.6 mL) were collected starting at 31.5 mL. The column was calibrated with molecular weight standards and the void volume determined with blue dextran. In some experiments, individual fractions from treated and untreated cells were concentrated using Amicon 10K Ultra-0.5 (Millipore, Carrigtwohill, Ireland) centrifugation filters and equal volumes were analyzed by E-PAGE Western blot and probed as described above.

### DARTS assay

The Drug Affinity Responsive Target Stability (DARTS) assay was optimized and used to assess protease protection from thermolysin as previously described [27,28]. KU174 was tested for protease protection using recombinant Hsp90α where a 25 μM concentration of each drug was used to treat 1 μg of recombinant Hsp90α for 15 min on ice. Following drug treatment the samples were digested with ~600U thermolysin for 10 min at RT. The digestion reaction was stopped with 50 mM EDTA and samples were analyzed by SDS-PAGE and Western blot. In addition, the N-terminal inhibitors, 17-AAG and radicicol, were used as positive controls along with untreated and vehicle (DMSO) treated recombinant Hsp90α.

### Biotinylated KU-174 co-immunoprecipitation

Biotinylated KU-174 and KU-174 (-noviose) were prepared by synthesis of their corresponding 3-(6-hydroxy-3'-methoxy-[1,1'-biphenyl]-3-ylcarboxamido) derivatives followed by biotinylation with NHS-PEG_4_-biotin in DMF at room temperature in the presence of TEA. Biotinylated compounds were isolated by RP-HPLC followed by vacuum drying with structure confirmation by mass spectrometry. A total of 1000 pmol of biotinylated compound was added to 1 mg of PC3-MM2 native lysates or 1 μg recombinant Hsp90 per reaction. In some reactions binding was competed with excess ATP using a regeneration system consisting of 2 mM ATP, 10 mM creatine phosphate disodium salt, 3.5 U/mL creatine kinase and 0.6 U/mL inorganic pyrophosphatase. Samples were immunoprecipated at 4°C with continuous rotation for 4 - 16 hours followed by the addition 50 μL of Dynabeads^® ^M-280 Streptavidin magnetic beads (Invitrogen, Oslo, Norway). After 15 minute incubation, beads were magnetically separated and pellets washed 5X with wash buffer (PBS, 1% BSA, 0.1% DDM). Captured Hsp90 protein was released by boiling samples with 50 μL SDS sample buffer. A total of 15 μL was loaded on an e-PAGE gel (Invitrogen, Carlsbad, CA) and probed for Hsp90 as described above.

### Surface Plasma Resonance (SPR)

*SPR analysis of KU174 binding to *Hsp90β was purified from baculovirus infected Sf9 cells and immobilized to SensiQ SSOO COOH1 SPR sensor chips as described previously [18,19]. KU174 (25 μl), diluted in assay buffer containing 10 mM PIPES pH 7.4, 300 mM NaCl, and 2% DMSO was injected over the surface of the derivatized chip at a flow rate of 25 μL/min at 25°C at the indicated concentrations with binding measured with a SensiQ SPR instrument (ICX Nomadics). Curves were double referenced to subtract contributions of the buffer containing 2% DMSO to the response units. QDAT software (ICX Nomadics) was used to analyze the sensorgrams for the kinetics of binding (k_a_) and dissociation (k_d_) and the SPR binding curves to estimate the affinity of binding (K_d_).

### Cancer cell based Hsp90 dependent luciferase refolding assay

Luciferase refolding assay was performed in cells previously stably trandsduced with lenti virus carrying Luc2/mCherry genes. Briefly, cell pelletes were collected from 80-90% confluent flasks and resuspended in pre-warmed media (50°C) for approximately 6 minutes. This time and temperature was sufficient to denature the endogenous luciferase to less than 2% of the basal activity but was insufficient to decrease viability of cells (data not shown). Cells were then plated at a density of 50,000 cells/well in a 96 well white plate in the presence of inhibitors. After one hour, the extent of refolded luciferase was measured by the addition of a luciferin substrate solution and read on a Victor III luminometer set for 0.1 sec/well integration. Direct inhibtion of luciferase was analysed for each compound as previously described [29]. IC_50 _values were calculated from raw data plotted or normalized to control using a non-linear regression and sigmoidal dose response curves (GraphPad Prism).

### In-vivo orthotopic tumor studies

#### Rat prostate xenograft tumor model single dose study

Eight week old nude rats (Crl:NIH-*Foxn1*^*rnu*^, Charles River) were inoculated orthotopically with 1 × 10^6 ^PC3-MM2 cancer cells. The rats were allowed to develop significant tumor burden, approximately 60-70 days, after inoculation. Subsequently, a single dose study of KU174 or vehicle was administered (i.p.) to treatment groups of five rats and the animals were sacrificed by exsanguinations six hours after injection. Immediately following blood collection, the thoracic cavity was opened and the animal was perfused exhaustively with saline. Tumors were collected and tumor to plasma ratio determined by standard bioanalytical methods.

#### Rat prostate xenograft tumor model efficacy study

Subsequent to the single dose study, an *in-vivo *efficacy study with KU174 was conducted using NIH nude rats inoculated subcutaneously in the flank with 2 × 10^6 ^PC3-MM2 cancer cells. Tumors developed for eight days at which time twenty rats were randomized into four treatment groups (vehicle, 15, 25, and 75 mg/kg KU174). The average tumor volume between groups was equal to ~30.13 mm^3 ^using the formula L × W × H. Rats were to be dosed daily for 14 consecutive days (day 0) and tumor volumes measured three times per week. Following the third dose, one vehicle treated and two KU174 treated (75 mg/kg dose), therefore the dosing schedule was changed to every other day to allow 48 hours recovery between doses, in case this was a result of toxicity. The 15 and 25 mg/kg groups continued on a daily dosing schedule until the animals were sacrificed on Day 17 while the vehicle and 75 mg/kg treatment groups continued with doses every other day with the study ending on Day 25 with no further mortality or apparent gross toxicity. Data were analyzed as the median percent increase in tumor volume relative to the initial tumor volume and tissues were sent to a veterinarian pathologist for toxicity analysis (Xenometrics, Stillwell, KS). Animal experiments were carried out in the animal facilities of The University of Kansas Medical Center with strict adherence to the guidelines of the IACUC Animal Welfare Committee of KUMC (IACUC protocol # 2009-1837).

## Results

### KU174 exhibits broad activity across the NCI60 cancer cell panel

Human tumor cell lines from the NCI60 panel were used to assess KU174 activity across cancers. This screen revealed that KU174 exhibits broad activity across multiple cancer cell lines (Table 1). Notably KU174 appears to be particularly active across the melanoma cell lines and was also cytotoxic in the multi-drug resistant ovarian adenocarcinoma cell line (NCI/ADR-RES). In the prostate cancer cell lines, PC-3 and DU145, KU174 was cytostatic at the single dose of 10 μM with values of 0.46 and 51.79, respectively. Furthermore, testing of the LNCaP-LN3 androgen dependent prostate cancer cell line in anti-proliferative assays demonstrate a GI50 of 128 nM (data not shown). Based on previous publications in prostate cancer using an earlier analogue, F-4 [18], we chose to focus on the characterization of KU174 in the PC3-MM2 and LNCaP-LN3 cell-lines to further understand its mechanism of action and effects on Hsp90.

**Table 1:** KU-174 Activity Across NCI-60 Human Tumor Cell Lines

Leukemia		Renal Cancer		Melanoma		Colon Cancer	
CCRF-CEM	0.63	786-0	73.59	LOX-IMVI	9.59	COLO-205	0.63
HL-60(TB)	0.44	A498	83.68	MALME-3M	41.99	HCC-2998	42.97
K-562	23.12	ACHN	47.1	M14	-21.95	HCT-116	21.97
MOLT-4	-21.98	CAKI-1	71.91	MDA-MB-435	-17.38	HCT-15	32.2
RPMI-8226	-44.19	RXF-393	105.66	SK-MEL-2	41.67	HT29	66.64
SR	28.61	SN12C	63.5	SK-MEL-28	21.39	KM12	-45.47
		TK-10	14.99	SK-MEL-5	-73.95	SW-620	38.13
		UO-31	18.75	UACC-257	-39.68		
				UACC-62	-27.18		
NSC-Lung Cancer		CNS Cancer		Ovarian Cancer		Breast Cancer	

A549/ATCC	4.66	SF-268	43.3	IGROV1	28.24	MCF7	5.74
EKVX	66.17	SF-295	43.01	OVCAR-3	14	MDA-MB-231	81.66
HOP-62	0.11	SF-539	77.23	OVCAR-4	1.21	HS-578T	51.84
NCI-H23	-21.22	SNB-19	72.87	OVCAR-5	103.07	BT-549	24.27
NCI-H322M	69.89	SNB-75	81.1	OVCAR-8	-13.39	T-47D	-27.58
NCI-H460	25.82	U251	38.81	NCI/ADR-RES	-5.97	MDA-MB-468	9.61
NCI-H522	2.5			SK-OV-3	98.1		
Prostate Cancer							
PC-3	0.46						
DU-145	51.79						

NOTE: The numbers reported are from a single dose (10 μM) study and are displayed as growth relative to the no-drug control after 24 hours. Values between 0 and less than 100 indicate growth inhibition whereas values less than 0 (negative values) indicate cytotoxicity.

### KU174 exhibits relatively specific cytotoxicity, to cancer cells compared to normal renal cells

**Figure 1:**
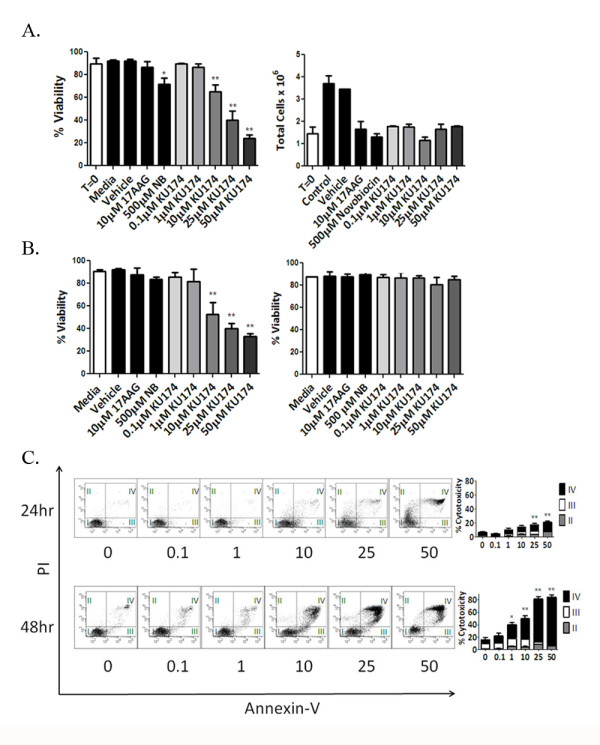
**KU174 mediated induction of selective cytotoxicity and apoptosis in PC3-MM2 prostate cancer cells**. Selective cytotoxicity was determined by trypan blue exclusion assay in PC3-MM2 cells (Figure 1A and 1B, *left panel*) at 24 and six hours, respectively. Normal RPTEC cells dosed for six hours did not show a loss of viability (Figure 1B, *right panel*). Furthermore, total PC3-MM2 cells (Figure 1A, *right panel*) were counted following 24 hours of treatment and compared to the number of cells plated at time zero (T = 0) demonstrating potent anti-proliferation of KU174 as low as 100 nanomolar. Further cytotoxicity studies were conducted by flow where viable cells (quadrant I) were compared to cells undergoing necrosis (quadrant II), early apoptosis (quadrant III) or late-stage apoptosis (quadrant IV) by measuring the percent Annexin V and propidium iodide (PI) staining of the parent population for PC3-MM2 cells (*Scatterplots *Figure 1C) following KU174 treatment. A bar graph of these data along with statistical analysis is shown (Figure 1C, *right panel*). Columns depict the mean ± SEM from three independent experiments (n = 3), * and ** indicates significant paired t-test *P value *of < 0.05 and < 0.01 respectively, compared to vehicle-treated control.

KU174 induced cytotoxicity in prostate cancer cells was assessed by trypan blue exclusion. PC3-MM2 (Figure 1) cells dosed with KU174 for 24 hours exhibited a dose-dependent decrease in viability ranging from 70-25% (Figure 1A, left panel). The parent compound NB, at 500 μM, resulted in a viability of ~75%; indicating KU174 manifests a 10-50 fold increase in potency compared to its parent molecule. No loss in cell viability was observed with 17-AAG at 10 μM which is consistent with previously published data demonstrating no cytotoxicity in either cell line at concentrations as high as 100 μM [18]. Comparing total cells to the time zero cell density revealed that 0.1 μM KU174 is as cytostatic as 10 μM 17-AAG (Figure 1A, right panel). These data show that KU174 is cytostatic at low relative concentrations (0.1 - 1 μM) and cytotoxic at higher concentrations (10 - 50 μM). In the LNCaP-LN3 cell line, the same trend was observed with respect to cytotoxicity with KU174 being approximately three to five fold more potent (data not shown). Furthermore, PC3-MM2 cells dosed with KU174 for only six hours (Figure 1B, left panel) resulted in a similar cytotoxic response as observed at 24 hours. Conversely, normal human renal proximal tubule epithelial cells (RPTEC) dosed with KU174 for 6 hours (Figure 1B, right panel) exhibited no loss in viability, providing evidence that KU174 is relatively selective for both prostate cancer cell lines. The RPTEC was selected as the normal cell line based on previous studies that Hsp90 inhibitors have a 100-fold lower affinity in normal cell lines compared to tumor cell lines [30].

Following 24 hour KU174 treatment, approximately 25-50% of the cells remain viable in the 10-50 μM range. Thus, the mode of cytotoxicity was examined between 24 and 48 hours of treatment by flow cytometry. PC3-MM2 cells were gated into four quadrants, identifying: viable (I), necrotic (II), early apoptotic (III), and late apoptotic (IV) cells. Figure 1C shows that KU174 treatment elicits two modes of action by inducing mostly necrosis within 24 hours as evidence by the cytotoxicity data above with little staining in quadrants III and IV. Furthermore, significant late stage apoptosis was observed on the remaining cells between 24 and 48 hours in a time and dose-dependent manner as evidence of the increase in number of cells in quadrant IV. Surprisingly, a majority of cells appeared in the late apoptotic quadrant (IV) with significantly fewer cells in the early apoptosis and necrosis quadrants (III, and II, respectively, Figure 1C, bar graphs). Likewise, a significant trend was observed in the LNCaP-LN3 cell line indicating these data are not unique to a single cell line (data not shown). These data demonstrate KU174 necrotic cytotoxicity between 6-24 hours and that cells remaining after the 24-hour treatment undergo dose-dependent apoptosis.

### KU174 results in a dose-dependent decrease in client proteins without a concomitant increase in Hsps

**Figure 2:**
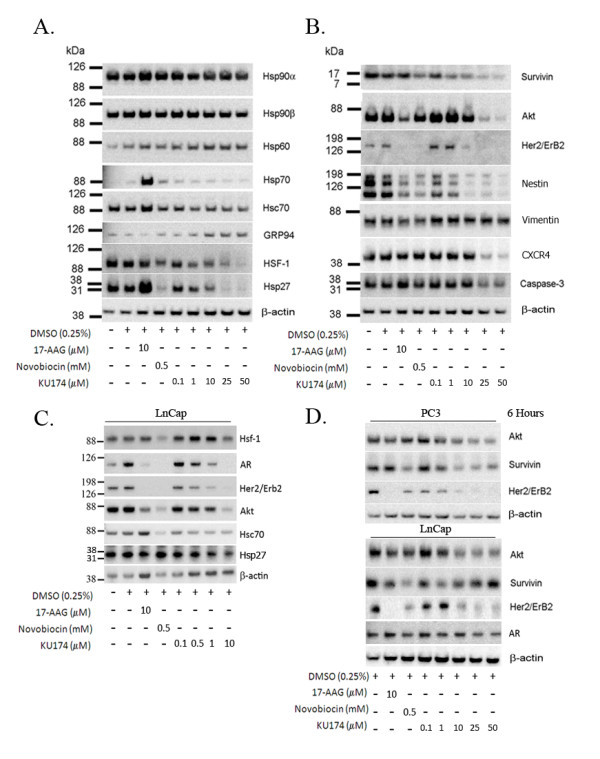
**Selective KU174 mediated client protein degradation in the absence of heat shock protein induction**. PC3-MM2 (Figure 2A and 2B) and LNCaP (Figure 2C) were examined for client protein degradation and the induction of heat shock proteins. 17-AAG demonstrates Hsp induction (Figure 2A) in PC3-MM2 while KU174 triggers degradation of Hsc70, HspHsp27 and HSF-1 with induction of GRP94 and HspHsp60 in PC3-MM2 cells. Notably, KU174 induced a dose-dependent decrease in several known proteins (Akt, Her2, nestin and CRCX4) that have been shown to play a role in the etiology of prostate cancer. A similar trend of client protein degradation was seen in LNCaP however this cell line was generally more sensitive to inhibitors (Figure 2C). Rapid client protein degradation within 6 hours was seen in both cell lines (Figure 2D).

A hallmark of Hsp90 inhibition is the selective degradation of Hsp90 dependent client proteins. Therefore, the level of expression of Hsp90 client proteins that are known to be associated with prostate cancer cell survival was examined [2,31-35] in prostate cancer cell lines. The potential of KU174 to trigger degradation of client proteins, effect Hsp modulators (HOP and HSF-1) and the assessment of heat shock protein induction were analyzed in the PC3-MM2 (Figure 2A-B) and LNCaP-LN3 (Figure 2C) following 24 hours of treatment. In both cancer cell-lines, KU174 demonstrated a dose-dependent reduction in Hsp (Hsc70 and Hsp27), HSF-1 and client proteins (survivin Akt, Her2, nestin, CXCR4 and caspase-3) whereas, a minimal effect was seen on these proteins in normal RPTEC cells (data not shown). Conversely, a modest induction of the ER chaperone, GRP94, and the mitochondrial chaperone, Hsp60 was observed with KU174 treatment (Figure 2), while no changes were observed in the expression of glucose-related protein 78 (GRP78)/Bip (data not shown). Importantly, KU174 at concentrations of five times higher than 17-AAG did not induce a significant heat shock response. Conversely, the N-terminal inhibitor 17-AAG caused a robust heat shock response inducing pro-survival Hsp70 and Hsp27 proteins in PC3-MM2 cells. Interestingly, since KU174 causes cytotoxicity as early as six hours, it can be hypothesized that client protein should correspondingly be degraded at this time point. In both prostate cancer cell lines, client protein degradation was observed which supports Hsp90 inhibition as the mechanism of cell death (Figure 2D).

### Analysis of native chaperone complexes by Blue Native-PAGE (BN-PAGE) and Size Exclusion Chromatography (SEC)

**Figure 3:**
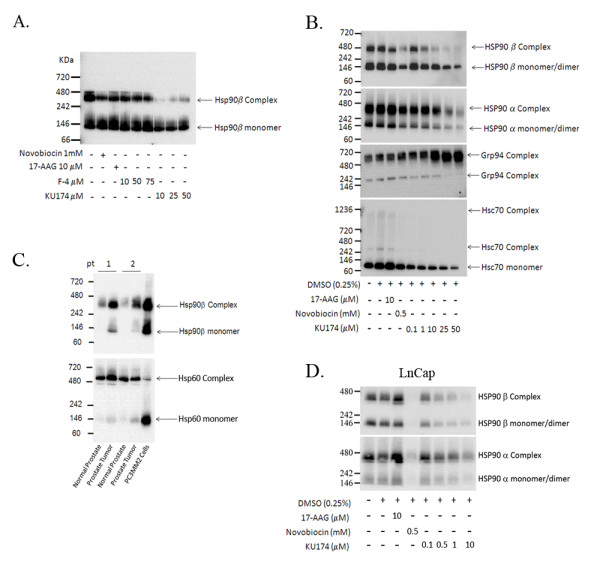
**Analysis of native Hsp90 complexes in PC3-MM2 and LNCaP cells by Blue Native Western blot and identification of the Hsp90 complex from human tissue**. Native Hsp90 complexes were identified by Native Western blot (Figure 3A). 17-AAG, novobiocin and F-4, an early novobiocin analog, had little effect on the Hsp90β complex. Conversely, cytotoxic concentrations of KU174 resulted in potent disruption of the Hsp90β complex (Figure 3A). Dose-dependent effects on Hsp90β, Hsp90α, GRP94 and Hsc70 native complexes were determined following treatment of cells KU174 along with novobiocin and 17-AAG (Figure 3B). Hsp90β complex was also observed in human cancer tissue from two patient samples but to a lesser extent in their normal adjacent tissue (Figure 3C). Hsp60 served as a loading control showing relative equal loading across the patient samples. KU174 also exhibits activity in the androgen dependent cell line LNCaP resulting in Hsp90 complex disruption (Figure 3D).

**Figure 4:**
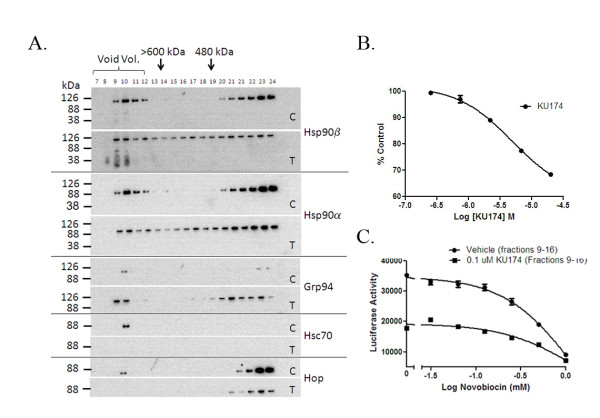
**Native Size Exclusion Chromatography (SEC) fractionation of Hsp90 complexes and inhibition of Hsp90 dependent luciferase refolding activity**. Vehicle or 25 μM KU174 treated (24h) SEC fractions were probed by SDS-PAGE Western blot. For each protein in Figure 4A, a vehicle control treated (Figure 4A, *panel C*) and a KU174 treated (Figure 4A, *panel T*) are depicted. Pooled vehicle fractions (9-16) showed the ability to refold thermally denatured luciferase and this activity was inhibited by KU174 (Figure 4B). In a follow-up study, PC3-MM2 cells treated with or without KU174 (0.1 μM) were fractionated by SEC and fractions 9-16 of each sample were pooled and chaperone activity was tested as a function of luciferase refolding (Figure 4C). The chaperone refolding activity of 0.1 μM KU174 treated samples is reduced to ~50% of vehicle *(see left axis segment) *and was further inhibited by novobiocin. These data suggest KU174 directly inhibits Hsp90 complex refolding activity.

Hsp90 functions as part of a large multiprotein complex and therefore, inhibition of Hsp90 may lead to disruption of these complexes. In order to study this process BN-PAGE Western blot studies were performed that allowed to the characterization of native chaperone complexes. In these studies, a robust disruption of an Hsp90|3 specific complex (Figure 3A) was observed, while NB, 17-AAG, and F-4 (a less potent early novobiocin analog) only moderately disrupted the complex. Furthermore, KU174's effect on various other native chaperone complexes was assessed; including Hsp90α, Hsp90β, GRP94 and Hsc70. These complexes resolved at a relative MW of 400 kDa for Hsp90α and Hsp90β, while GRP94 complexes migrated near 720 kDa and 242 kDa with Hsc70 resolving mainly as a monomer under these native conditions (Figure 3B). Differential disruption of Hsp90α and Hsp90β complexes was observed with C-terminal Hsp90 inhibitors, NB and KU174 (Figure 3B) with little to no effect by the N-terminal inhibitor 17-AAG. A drug-dependent increase in GRP94 complexes along with a decrease in HscHsc70 monomer and complex was observed with KU174 but not with 17-AAG. A potential criticism of the data in Figure 3A-B is that these complexes are a product of *in-vitro *culture conditions and not physiologically relevant. To address this issue, two prostate cancer patient samples were analyzed along with the corresponding normal adjacent tissue and the mitochondrial chaperone Hsp60 was used as a loading control. These results yielded similar Hsp90 native complexes compared to those observed in the PC3-MM2 cell-line (Figure 3C). Similar results were also observed with the androgen dependent cell line LNCaP-LN3, however these cells were generally more sensitive to KU174 in terms of dissociating the native Hsp90 complexes (Figure 3D). To further investigate these complexes, native Hsp complexes were fractionated by SEC and analyzed by SDS-PAGE Western blot. Chaperone complexes were identified containing Hsp90β, Hsp90α, and GRP94, all of which appeared to shift in MW following KU174 (25 μM) treatment compared to vehicle treated cells (Figure 4A). With respect to Hsp90α and Hsp90β, these observations, taken in context with the apparent disruption of the ~400 kDa complex observed in BN Western blots (Figure 3B), suggests that these higher MW complexes were unable to enter the BN gel or did not resolve into distinct bands and therefore gave the impression in BN gels of a decreased complex at 400 kDa. Significant Hsps were also detected in the column void volume (fractions 7-12). Interestingly, Hsp90β eluted within the void volume and showed degradation that was not observed in the Hsp90α blot, raising the potential that Hsp90β is degraded *in situ *with bound client proteins. Additionally, Figure 4A demonstrates that the co-chaperones HOP and Hsc70 co-elute within the void volume (fraction 10) in vehicle but not with KU174 treated samples providing evidence that KU174 disrupts the binding or stability of these co-chaperones in complex with Hsp90. The functionality of these higher MW chaperone complexes was further assessed by subjecting the native fractions to a novel luciferase refolding assay adapted from the widely used rabbit reticulocyte assay developed by Matts and colleagues [29]. PC3-MM2 cells dosed with vehicle or 0.1 μM KU174 for 24 hours were lysed and fractions 9-16 collected by SEC. The chaperone activity from the pooled fractions of each sample was tested as a function of luciferase refolding as described in Materials and Methods. Vehicle fractions 9-16 showed luciferase refolding activity which could be inhibited in a dose-dependent manner by KU174 (Figure 4B). Furthermore, cells treated with 0.1 μM KU174 for 24 hours showed a decrease in activity by approximately 50% compared to vehicle (Figure 4C). The refolding activity for both vehicle and treated fractions was further inhibited in a dose-dependent manner with novobiocin. These data suggest that Hsp90 complexes eluted within SEC fractions 9-16 are active and retain chaperoning ability as measured by their refolding of thermally denatured luciferase.

### DARTS Assay of KU174 binding to Hsp90

**Figure 5:**
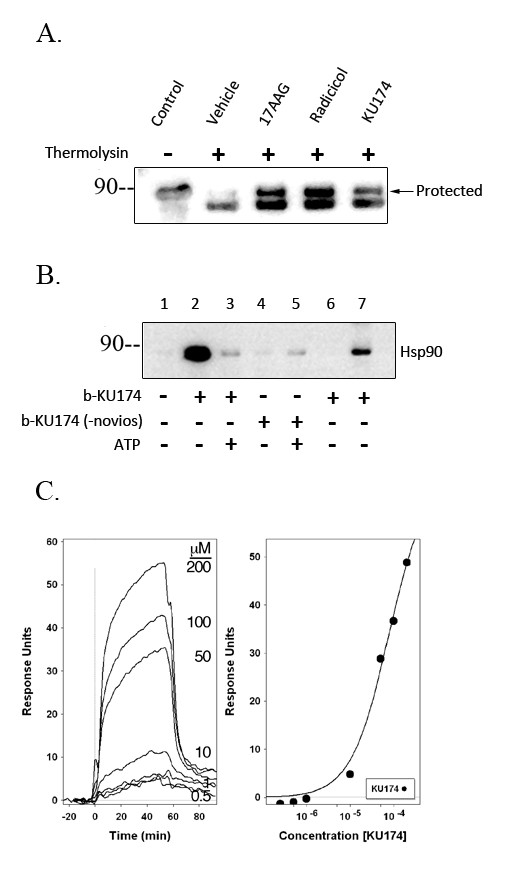
**Analysis of the binding of KU174 to Hsp90**. Drug Affinity Responsive Target Stability (DARTS) was used to test the specificity of KU174 to Hsp90. Recombinant Hsp90 was incubated with 25 μM of KU174, 17-AAG, radicicol or vehicle, followed by digestion with thermolysin and analysis by SDS-PAGE Western blot for protection of Hsp90 protein (Figure 5A). Upper band is protected from proteolysis by both N-terminal and C-terminal Hsp90 inhibitors. Biotinylated KU174 (b-KU174) but not the inactive analogue (-noviose) bound with sufficient affinity to immunoprecipitate Hsp90 in native PC3-MM2 cell lysates (Figure 5B). Importantly, binding was prevented with excess ATP. KU174 was injected over Hsp90β immobilized to the surface of a SPR sensor chip at concentrations of 0.25, 0.5, 1.0, 10, 50, 100, and 200 μM as described under "Materials and Methods". Sensorgrams of KU174 binding to Hsp90β are shown (Figure 5C, *left*) and a concentration dependent binding curve for the interaction of KU174 with Hsp90β (Figure 5C, *right*).

Binding of a drug/ligand to its target protein results in conformational changes and proteolytic stabilization of the protein by reducing sensitivity to proteases [27,36,37]. Similar in concept to DNase protection assay [38-40], or protease protection assay, Drug Affinity Responsive Target Stability (DARTS) [27,28] was used to test the specificity of KU174 for Hsp90. Recombinant Hsp90 was incubated with 25 μM of KU174, 17-AAG, radicicol or vehicle, followed by digestion with thermolysin and analysis by SDS-PAGE Western blot for protection of Hsp90 protein. KU174 along with the known Hsp90 N-terminal inhibitors, 17-AAG and radicicol, protected Hsp90 from degradation as evident by the upper band (Figure 5A) that is apparent in the control (no thermolysin lane), but absent in the vehicle treated lane that received thermolysin. These data demonstrate the direct binding of KU174 to Hsp90.

### Co-immunoprecipitation of biotinylated KU174 and Hsp90

In order to further support that KU174 binds Hsp90, biotinylated KU174, along with an inactive analogue lacking a critical noviose sugar, was used in co-immunoprecipitation experiments. Using PC3-MM2 cell lysates in the presence or absence of ATP (Figure 5B), biotinylated KU174 (b-KU174) but not the inactive analogue (-noviose) bound with sufficient affinity to immunoprecipitate Hsp90 and that binding is prevented with excess ATP. While it is unclear whether the ATP is competing directly at the C-terminal site or is acting allosterically by binding to the N-terminus and thus preventing accessibility at the C-terminal pocket, this data demonstrates that KU174 is binding directly to Hsp90.

### Surface Plasma Resonance (SPR)

In order to further characterize KU174 as a direct Hsp90 inhibitor, the binding of KU174 to Hsp90 was analyzed by surface plasmon resonance (SPR) spectroscopy (Figure 5C). The kinetics of binding and dissociation were reliably fitted to a pseudo-first order model for a 1:1 interaction with the k_a _and k_d _calculated to be 1.04 × 10^3 ^(M^-1.^sec^-1^) and 0.098 (sec^-1^), respectively. The K_d _estimated from the fitting of the binding curve (78 μM ± 7 s.e.) was in close agreement with the K_d _estimated from the ratio of the dissociation and association constants (94 μM ± 4 s.e.). In comparison, the k_a _and k_d _for the binding of novobiocin to Hsp90 were 211 (M^-1.^sec^-1^) and 0.23 (sec^-1^) (calculated K_d _of 1.1 mM ± 0.4 s.e), with a K_d _calculated from the binding curve of 0.86 mM ± 0.02 s.e.). Thus, the SPR analysis of the interaction of KU174 with Hsp90 indicated the compound bound directly to the purified recombinant protein with an affinity approximately 12-fold higher than NB.

### Cancer cell based Hsp90 dependent luciferase refolding assay

**Figure 6:**
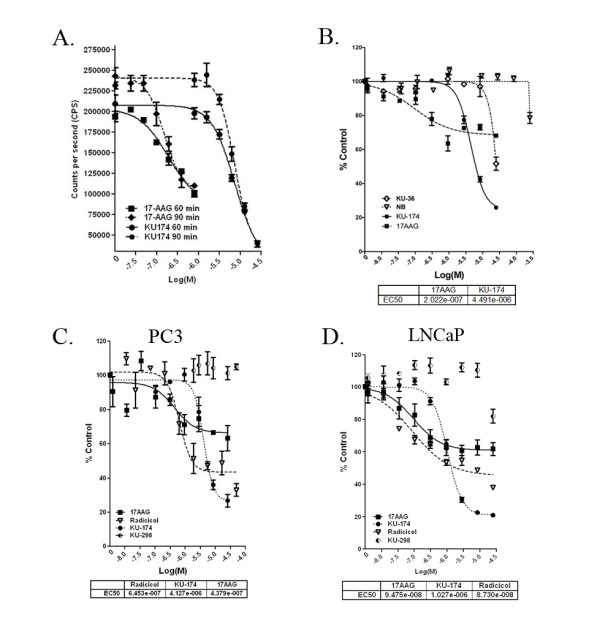
**Cancer cell based Hsp90 dependent luciferase refolding assay**. The extent of thermally denatured luciferase refolding in PC3-MM2 in the presence of N-terminal or C-terminal Hsp90 inhibitors was tested at 60 and 90 minutes (Figure 6A). The parent compound NB and an earlier, less potent, analogue F-4, along with KU174 and 17AAG was subsequently tested for their ability to inhibit luciferase refolding over 60 minutes (Figure 6B). Next we compared a second N-terminal inhibitor, radicicol, and an inactive novobiocin analog, KU298, determined in our laboratory to not bind Hsp90 as an additional positive and negative control for this assay, respectively (Figure 6C). Similar results were also seen in the androgen dependent cell line LNCaP; however, 17AAG, radiciol and KU174 exhibited increased potency (Figure 6D).

Direct inhibition of the Hsp90 protein folding machinery was assessed using a cancer cell-based luciferase refolding assay developed in our laboratory. Previously, the Hsp90 luciferase-based refolding assay has been validated using rabbit reticulocyte lysates. However, there remains concern whether the presentation of Hsp90 complexes within these lysates are physiologically relevant in cancer. Several lines of evidence suggest that Hsp90 is present in cancer cells as part of a large macromolecular complex and therefore drugs that target Hsp90 activity should be engineered towards binding Hsp90 within its physiologically relevant cancer cellular environment. Based on the aforementioned limitations using rabbit reticulocyte lysates, a cell-based luciferase assay was optimized using both *N-*terminal and *C-*terminal Hsp90 inhibitors in prostate cancer cell lines (Figure 6A-D). The extent of luciferase refolding in PC3-MM2 in the presence of N-terminal or C-terminal Hsp90 inhibitors was evaluated at 60 and 90 minutes. Both classes of Hsp90 inhibitors demonstrated similar EC_50 _concentrations at 60 and 90 minutes (Figure 6A) with 17-AAG being more potent. Since a 60 minute refolding experiment resulted in a significant increase in luciferase activity and good signal to noise, all subsequent experiments were performed at this time point. In order to demonstrate assay performance and accuracy, the parent compound NB and an earlier, less potent analogue, F-4 was compared to KU174 and 17AAG. As expected, NB and F-4 resulted in right shifted dose response curves relative to KU174 with NB showing minimal activity (Figure 6B). Subsequently, a second N-terminal inhibitor, radicicol, and an inactive novobiocin analog determined in our laboratory to not bind Hsp90, KU298, were analyzed in this assay as additional positive and negative controls, respectively. In this experiment, radicicol demonstrated an EC_50 _value comparable to 17-AAG, while as expected KU298 was inactive, further supporting the specificity of this assay for Hsp90 inhibition (Figure 6C). Finally, to compare this assay across prostate cancer cell lines, the ability of Hsp90 inhibitors to inhibit luciferase refolding was examined in an LNCaP-LN3 luciferase expressing cell line (Figure 6D). In agreement with our previous results, these compounds inhibited Hsp90 dependent luciferase refolding with increased potency (EC_50_) when comparing EC_50 _values between cell lines, a trend that has also been observed in other functional assays. Overall, these data demonstrate a novel approach to determine on-target Hsp90 inhibition using a functional assay in an intact cancer cell milieu.

### In-vivo preclinical proof-of-concept studies

**Figure 7:**
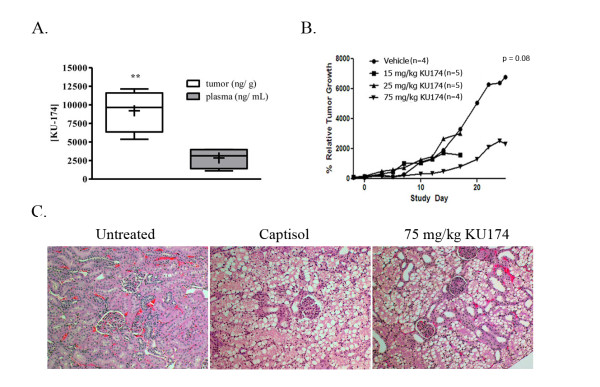
**In-vivo efficacy and selective tumor accumulation of KU174 in rat prostate tumor models**. Six hours following a single i.p. dose, the tumor to plasma ratio of KU174 was determined to be ~3.6:1 suggesting selective tumor accumulation (Figure 7A, **p value < 0.01). Median percent increase in tumor volume relative to the initial tumor size from a pilot study in a rat PC3-MM2 tumor xenograft dosed with KU174 (15, 25, and 75 mg/kg) Tumor growth inhibition was evident between vehicle (n = 4) and the 75 mg/kg group (** p = 0.08, n = 4) (Figure 7B). Microscopic examination of kidneys from both vehicle (captisol) and 75 mg/kg KU174 treated animals showed prominent vacuolization compared to untreated (Figure 7C) indicating captisol resulted in some degree of toxicity.

Initially, pilot pharmacokinetic (PK) studies of KU174 were conducted in the mouse (data not shown) and revealed extensive metabolism and clearance preventing the use of this species for efficacy studies as effective concentrations of drug could not be achieved at the site of action (tumor). Therefore, KU174 was initially tested in the rat PC3-MM2 xenograft tumor model in a single dose pilot PK study to ensure that effective concentrations could be reached in the tumor prior to conducting a multi-dose efficacy study. A KU174 tumor to plasma ratio of 4:1 was achieved six hours after a single i.p. administration of 75 mg/kg suggesting selective retention (Figure 7A). The concentration of KU174 in the tumor correlated to ~17 μM, assuming a gram of tissue is equal to one milliliter, at this time point, which was believed to be sufficient enough to observe a pharmacodynamic response based on our *in vitro *data. Following this single dose study, a multi-dose efficacy study was conducted using a rat PC3-MM2 xenograft tumor model so that tumor volume could be monitored over time. In this study, KU174 (15, 25, 75 mg/kg) was administered by i.p. injection in tumor burden rats as described in materials and methods. When the median percent increase in tumor volume was analyzed relative to the initial tumor volume, a consistent trend was evident and demonstrating a decrease in tumor size in the 75 mg/kg KU174 treated animals (p = 0.08, n = 4) (Figure 7B). Additionally, one animal was lost from the vehicle and 75 mg/kg treatment group during the course of the study. To rule out toxicity from either the vehicle or KU174, major organs (liver, kidney, heart, lungs, and prostate) were collected from all animals remaining at the end of the study. The tissues were examined by a veterinary pathologist for the presence of KU174 toxicity. Treatment associated microscopic lesions were noted in the heart, kidney, liver, and lung for both vehicle and KU174 treated groups which was concluded to result from vehicle. The severity of the morphological changes by tissue were kidney > lung > liver > heart and it was concluded these effects were caused by vehicle administration. Microscopic examination of kidneys from both vehicle (captisol) and 75 mg/kg KU174 treated animals showed prominent vacuolization compared to untreated (Figure 7C). In summary, KU174 demonstrates a significant reduction in tumor volume based on this pilot study with no signs of apparent toxicity; however, there was evidence of acute vehicle toxicity which was most evident in kidneys.

## Discussion

Since 1995, when the first Hsp90 inhibitor was shown to demonstrate antitumor efficacy in mouse xenograft tumor models, there has been considerable effort focused on the development of Hsp90 inhibitors for the treatment of cancer. To date, there have been minor differences reported between N-terminal or C-terminal Hsp90 inhibitors. We recently reported that the novobiocin analogue, F-4 induces client protein degradation with minimal Hsp90 induction in androgen dependent and independent prostate cancer cells [18]. These were some of the first pieces of evidence that showed C-terminal inhibitors to possess a unique pharmacology when compared to N-terminal inhibitors. A hallmark of N-terminal Hsp90 inhibition is the induction of Hsps (Hsp27, Hsp70 and to a lesser extent Hsp90) mediated through HSF-1 transcriptional activation of the heat shock response element (HSE). This is of significant concern because clinical resistance has been attributed to the induction of pro-survival Hsps [11,41,42]. As a result, targeting Hsp70 and Hsp27 has become an attractive paradigm for the prevention of resistance with future Hsp90 inhibitors. Herein, the development of a more potent C-terminal Hsp90 inhibitor, KU174 is described, which not only results in client protein degradation in androgen dependent and independent cell lines but also causes concomitant reduction of Hsc70, Hsp27 and HSF-1 without Hsp70 induction. Notably, these client proteins, heat shock proteins and Hsp90 modulators are all novel drug targets. In addition, some client proteins (CXCR4 and survivin) were degraded by KU174 but not 17-AAG suggesting inhibition of the N-terminal and C-terminal sites effect different subpopulations of proteins. Thus, KU174 elicits a combinatorial attack on numerous drug targets in prostate cancer cells resulting in potent cytotoxicity as early as six hours that is relatively selective for tumor cells versus normal cells (Figure 1B).

The induction of GRP94 at the total protein level (Figure 2A) and with respect to native complexes (Figure 3D) was a surprising result. GRP94 up-regulation has been associated with ER stress but is also correlated with increased tumor immunogenicity [42]. Thus, the significance of GRP94 induction with KU174 is unclear and will require further investigation. To date, there has been little focus on the different biological activities manifested by Hsp90 inhibitors with regard to the Hsp90α and Hsp90β isoforms and their respective native complexes. In this study for the first time, we reveal that a C-terminal Hsp90 inhibitor can induce a major 400 kDa Hsp90 native complex into higher MW supercomplex which seems to be relatively more selective for Hsp90β. Interestingly, the concentrations at which this effect is observed corresponds nicely with our cytotoxicity data (Figure 1A). Furthermore, KU174 induced Hsp90β degradation with no effect on Hsp90α (Figure 4A), suggesting a possible isoform selective response to chaperone inhibition. One hypothesis is that the apparent KU174 induced shift to higher MW complexes is a result of increased Hsp90 inhibited chaperone complexes containing unfolded client proteins. Thus, it's plausible that as unfolded client protein becomes ubiquitinated, Hsp90β is collateral damage and is degraded *in-situ *with its bound client protein. In support of this, recent preliminary data demonstrates the induction of polyubiquitinated proteins that co-elute with the partially degraded Hsp90β (data not shown).

Functionally, Hsp90 complexes isolated by SEC from KU174 treated cells can refold denatured luciferase but to a lesser extent compared to vehicle treated prostate cancer cells. Although further characterization and functional studies are required on the lower relative MW SEC fractions, these data suggest that the large (>600 kDa) Hsp90 complex is a functional chaperone complex and when inhibited by a C-terminal Hsp90 inhibitor leads to the partial degradation of Hsp90β but not Hsp90α (data not shown). Collectively, the direct binding of KU174 to recombinant Hsp90 is demonstrated using DARTS, and SPR experiments as well as biotinylated KU174 that co-immunoprecipitates Hsp90 from tumor cell lysate, which can be eluted in an ATP-dependent manner. Functionally, the inhibition of Hsp90 complexes in tumor cell lysate and intact cancer cells is shown using the Hsp90 dependent luciferase refolding assay. Collectively, these data demonstrate direct on-target inhibition of Hsp90 at concentrations that correlate to cytotoxicity, client protein degradation and disruption of Hsp90 complexes by SEC and BN Western blot.

Pilot *in *vivo efficacy studies were conducted and while there are limitations of this study, the results are encouraging, especially in light of the rather aggressive nature of PC3-MM2 tumors and the fact there has been little success in establishing human prostate tumor xenograft models in the rat. Collectively, these data demonstrate the *in-vivo *efficacy of KU174 in an aggressive androgen independent prostate cancer cell-line. Larger *in-vivo *efficacy studies to determine more precisely the effectiveness of KU174 in orthotopic and metastatic PC3-MM2 tumor models in rat are currently being designed.

## Conclusions

In this study, the biological differences between the N and C-terminal Hsp90 inhibitors, 17AAG and KU174, are highlighted in prostate cancer cells. Most notably, the C-terminal Hsp90 inhibitor, KU174, elicits its anti-cancer activity without inducing a HSR, which is a detriment associated with N-terminal inhibitors. Additionally, a novel approach to examine inhibition of Hsp90 complexes was developed using BN Western blot, SEC and luciferase refolding assays in intact cancer cells. These new approaches, along with newer assays being developed in our lab to address the issues of Hsp90 isoform specificity and selectivity, give us valuable mechanisms to investigate the development of future C-terminal Hsp90 inhibitors. KU174 and other C-terminal Hsp90 inhibitors are currently in early preclinical development for a number of cancers, in addition to prostate. We continue to focus on improving the potency and pharmacokinetics of these compounds to further evaluate *in-vivo *efficacy and identify a lead candidate for clinical trials.

## Abbreviations

17-AAG: 17-allylamino-17-demethoxygeldanamycin; BN: Blue-Native; DDM: n-dodecyl beta-D-maltoside; DMSO: Dimethylsulfoxide; EDTA: Ethylenediaminetetraacetic acid; GRP94: Glucose-regulated protein of 94 kDa; HOP: Hsp-organizing protein; HSF-1: Heat shock factor 1; Hsp(s): Heat shock protein(s); HSR: Heat-shock response; Hsp90α: Heat shock protein 90 alpha isoform; Hsp90β: Heat shock protein 90 beta isoform; IP: Intraperitoneal injection; MEM: Minimal essential media; NB: Novobiocin; RPTEC: Normal human renal proximal tubule epithelial cells; SEC: Size exclusion chromatography; SPR: Surface Plasma Resonance

## Competing interests

The authors declare that they have no competing interests.

## Authors' contributions

Conception and design: GV, JE, RR and JH, Cell viability and flow cytometry: JE and AD, Westen blot studies: JE, PM, MS, and SH, Blue native Western studies: JE and TS, Size exclusion chromoatography studies: JE and XZ, DARTS and Co-immunoprecipitation assays: JE and RR, SPR binding studies: JM and BM, Cell based luciferase assay: JE and TS, Animal studies: GV, ID, and RR, Synthesis and scale-up of KU-174: BB, AD, RR, Data Analysis and interpretation: JE and GV, Manuscript writing: JE, GV, JH, and BB, Final approval of manuscript: GV. All authors have read and approved the final manuscript.

## Pre-publication history

The pre-publication history for this paper can be accessed here:

http://www.biomedcentral.com/1471-2407/11/468/prepub
